# Granulomatosis with Polyangiitis Overlapping with IgG4-Related Disease

**DOI:** 10.1155/2022/2360060

**Published:** 2022-09-02

**Authors:** Aureliano Pistone, Muhammad Soyfoo

**Affiliations:** Department of Rheumatology, Cliniques Universitaires de Bruxelles – Hôpital Erasme, Université Libre de Bruxelles (ULB), Bruxelles, Belgium

## Abstract

IgG4-related disease and granulomatosis with polyangiitis share several features as well as the presence of ANCA antibodies and serum IgG4 immunoglobulins. It is often difficult to distinguish between two entities. We hereby report the case of a patient portraying the clinical conundrum with clinical and biological features of the two diseases.

## 1. Introduction

Granulomatosis with polyangiitis (GPA), previously known as Wegener's granulomatosis is an ANCA-associated vasculitis mainly involving the lungs and kidneys [1, 2]. This disease involves small to medium size blood vessels (more frequently small-sized vessels) with a reported annual incidence of 0.4 to 11.9 cases per million. GPA can affect people from all ethnic origins with predominance for white populations. The male to female ratio is 1 : 1. The cause of GPA is still not known. Although almost every small vessel can be affected by GPA, the upper and lower respiratory system and the kidneys are frequently involved. The disease can present as a continuous spectrum of clinical manifestations ranging from mild to severe organ-threatening conditions jeopardizing the lives of patients. One of the hallmarks of GPA is the presence of the antineutrophil cytoplasmic antibody (ANCA) in nearly 90% of cases, and up to 75% of cases are associated with PR3 (proteinase 3) ANCA [3]. Tailored and adapted targeted therapy is cardinal to decrease the burden of disease while increasing survival [4, 5].

IgG4-related disease (IgG4-RD) is an immune-mediated inflammatory disorder affecting several organs with a wide spectrum of clinical manifestations. The affected organs share several clinical and biological characteristics such as tumor-swelling like of organs, lymphoproliferative infiltration, and a certain extent of fibrosis described as “storiform fibrosis.” The pathological hallmark of IgG4-RD is lymphoplasmacytic infiltrates containing IgG4+ plasma cells of the affected tissues [2, 6]. Serum IgG4 levels increased in more than two-thirds of patients. The two main manifestations of this disease are autoimmune pancreatitis and sclerosing sialadenitis, but other organs can be affected. Cholangitis, dacryoadenitis, retro-peritoneal fibrosis, mediastinal fibrosis, tubulointerstitial nephritis, hepatitis interstitial pneumonia, thyroiditis, prostatitis, and gastritis have also been reported [7]. This disease generally responds well to glucocorticoids and immunosuppressants and more specifically in the absence of significant fibrosis [8].

Because of several similar clinical signs and the presence of ANCA antibodies and serum IgG4 immunoglobulins, differentiating IgG4-RD from GPA is not always easy. Furthermore, increased serum concentration of IgG4 has been observed in GPA as well as the presence of infiltration of IgG4 positive cells in tissues in patients with GPA. Currently, the existence of an overlap between the two diseases is not clearly certified [9–12]. We hereby present a case illustrating this situation.

## 2. Case Presentation

**Figure 1:**
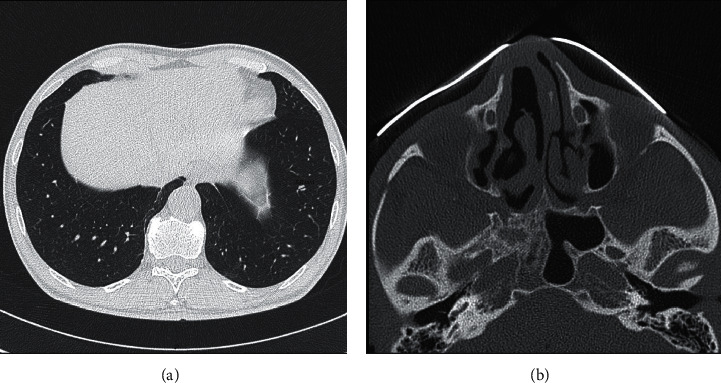
Computed tomography of lungs showing several micronodules (a) and that of sinus showing pansinusitis (b).

**Figure 2:**
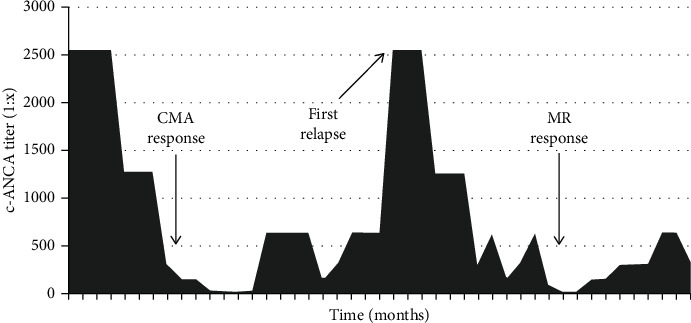
Evolution of c-ANCA titers over time. CMA: cyclophosphamide and methylprednisolone-based treatment followed by azathioprine. MR: methylprednisolone and rituximab-based treatment. This figure represents the evolution of c-ANCA titers over time. Peaks correspond to episodes of clinical relapses, and the falls correspond to improvements after treatment.

A 60-year-old patient was admitted to our hospital because of inflammatory syndrome, polyarthralgia, and weight loss. A physical examination showed only pain in the heels and the first metatarsophalangeal joint as well as hypoesthesia of the left foot. The significant biological results showed nonidentified c-ANCA (non-PR3 and non-MPO) with a titer of 1 : 2560 as well as a C-reactive protein of 99 mg/L, and an erythrocyte sedimentation rate of 67 mm/h. The renal function was in the normal range values. The thoracic computed tomography (CT) scan showed several bilateral nonspecific micronodules (Figure 1(a)), and the positron emission tomography computed tomography (PET-CT) scan showed moderately hypermetabolic retroperitoneal and inguinal lymphadenopathy. A sinus CT scan was performed and showed pansinusitis (Figure 1(b)). A biopsy of inguinal adenopathy was performed in favour of reactive inflammatory nodes with no signs of malignancy. The tuberculosis QuantiFERON ELISA test was positive, but the culture and polymerase chain reaction of the biopsied node were negative. The patient later developed arthritis in the ankle confirmed by IRM, and sinus CT confirmed the presence of pansinusitis. The diagnosis of GPA was made on the basis of high c-ANCA titers (non-PR3 and non-MPO), bilateral pulmonary nodules, and pansinusitis, and treatment with cyclophosphamide and methylprednisolone was administered, followed by azathioprine. This treatment was combined with isoniazid for tuberculosis prophylaxis. The patient presented a good clinical evolution with normalisation of the inflammatory syndrome and a significant decrease in c-ANCA (Figure 2). Five years later, the patient's clinical condition deteriorated with asthenia, weight loss, and abdominal pain. There was an increased inflammatory syndrome as well as increased ANCA titers (variation of titers is shown in Figure 2). The PET-CT scan was not in favour of a vasculitis relapse. The node biopsy performed five years ago was retrospectively reviewed for evaluation of IgG4. The histology of the lymph node was described as an inflammatory node with extensive plasmacytosis as depicted with CD138 immunostaining. The infiltrate showed 50 IgG4 positive plasma cells per high-power field. A serum IgG4 assay was performed and very slightly increased at 1.351 g/L (*N* < 1.350). The abdominal CT image did not show retroperitoneal fibrosis despite the presence of bilateral uretero-hydronephrosis without objectified obstacle. A possible diagnosis of IgG4-related disease (IgG4-RD) was then carried out, but GPA diagnosis was not completely excluded. The patient was treated by intravenous methylprednisolone and rituximab with a good clinical and biological evolution. Three years later, he developed another relapse with distal digital ischemia of the foot, multiplex mononeuritis, and external popliteal sciatica deficit, rather in favour of GPA with a BVAS score of 6 (Birmingham Vasculitis Activity Score). Afterwards, the patient was treated only with mycophenolate mofetil (2 g/day) without any relapse.

## 3. Discussion

GPA and IgG4-RD are today two better known diseases. GPA is an ANCA-associated vasculitis that can be defined by the 1990 ACR criteria and the 2012 Chapel Hill Consensus Conference. Recently, an Iranian team proposed a new algorithm called 2017 ACR/EMA Revised Criteria for Very Early Diagnosis of GPA [13].

IgG4-RD diagnosis can be based on the CDC according to the presence of organ involvement, the presence of IgG4/IgG ratio >40% abd the presence of high power fieds immunostaining for IgG4 more than 10. The diagnosis can be established as either possible, probable or definite [14]. Recently, Wallace et al. proposed the 2019 American College of Rheumatology (ACR)/European League against Rheumatism Classification Criteria for IgG4-Related Disease, which is not yet used in clinical practice [15].

Currently, the existence of an overlap between the two diseases is not clearly certified. Some authors believe that this association is possible and represent as such a particular phenotype. Organ involvement occurring in AAV patients could be related to IgG4-RD rather than to refractory granulomatous lesions, especially in GPA patients [9]. In this latter study, the authors identified some patients depicting clinical and/or histological clues pertaining to both diseases thereby raising the question of a potential overlap syndrome sharing common pathophysiological characteristics. We found only one case report of the patient suffering from GPA, IgG4-RD, and a small cell lung cancer [2].

On the other hand, there is even a case report of GPA mimicking IgG4-RD [16]. It is important to note that GPA biopsy can mimic IgG4-RD portrayed by inflammatory background [17]. It was demonstrated that IgG1 and IgG4 were the predominant subclasses of ANCA in patients with GPA. [18] In 2013, Chang et al. showed that 8/43 GPA patients had IgG4+ biopsy with the IgG4+ cells criteria (average IgG4+ cells greater than 30 per HPF with the ratio of IgG4+/IgG+ cells greater than 40%). However, all patients had histological characteristics of GPA like necrosis, necrotizing granulomas, abscesses, and vasculitis [19].

The association of GPA and IgG4-RD as a potential overlap syndrome with common pathophysiological mechanisms responsible for clinical and biological manifestations is therefore probable. However, both diseases have complex and still yet undeciphered pathological pathways. The link between the pathological role of ANCA and IgG4 in purporting damage in both diseases is still unknown. Certain lines of evidence underscore the role of *T* follicular helper cells that have increased in both diseases [9].

Our patient illustrates the difficulty to differentiate between these two diseases. In fact, the patient presented the characteristics of the two diseases. The presence of c-ANCA with pulmonary micronodules, pansinusitis, multiplex mononeuritis, and a decrease in ANCA with treatment is in favour of GPA. In 2009, the diagnosis was made following the consensus methodology for the classification of ANCA-associated vasculitis despite the presence of nonidentified c-ANCA [20]. Besides, cases of GPA with nonidentified ANCA were described in the literature [21]. However, the lymphadenopathy associated with a high level of IgG4 and node biopsy positive for IgG4 positive plasma cells are compatible with IgG4-RD. But we could not affirm the diagnosis of IgG4-RD if we apply the 2019 ACR criteria [15]. It is therefore difficult to confirm whether the patient has an overlap between GPA and IgG4-RD or GPA mimicking IgG4-RD.

## 4. Conclusion

This case illustrates well the possible coexistence of characteristics of ANCA vasculitis and IgG4-RD, as well as the difficulty to differentiate between the two diseases and above all, to affirm the presence of an overlap syndrome.
